# Supplementary material for: Link of Patient Care Outcome to Occupational Differences in Response to Human Resource Management: A Cross-Sectional Comparative Study on Hospital Doctors and Nurses in China
Source: Int J Environ Res Public Health. 2020 Jun 18;17(12):4379. doi: 10.3390/ijerph17124379 (PMC7344802; doi:10.3390/ijerph17124379)
Supplement: Supplementary file 1 [file ijerph-17-04379-s001.pdf]

Appendix 1: Sensitivity tests for HPWS measurements

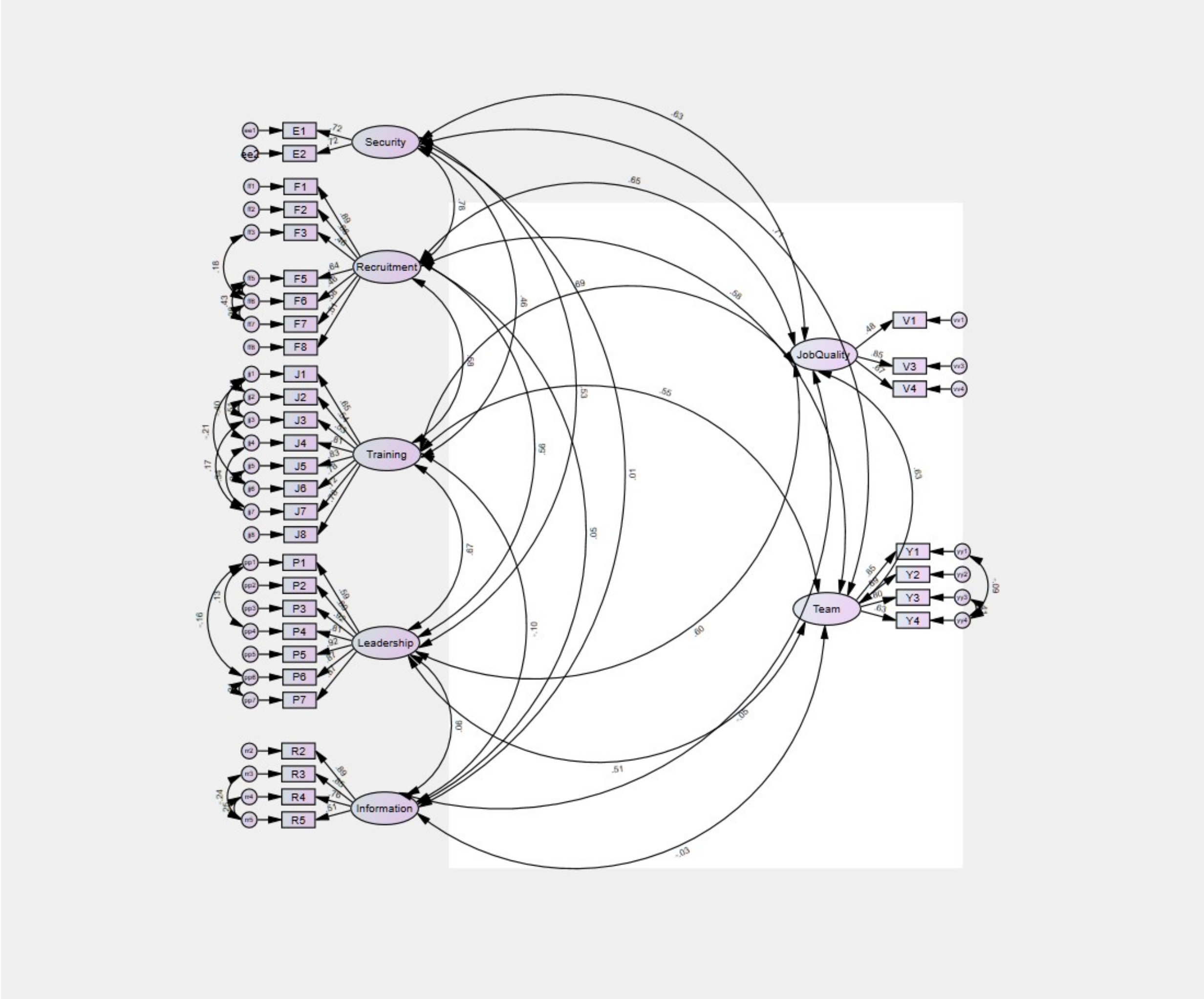

Figure A. Confirmation Factor Analysis on the HPWS measurements with seven factors

Model fit: CFI=0.919, TLI=0.907, RMSEA=0.060, Chi-square=1440.369 (p<0.001)

Recruitment (y2)

A1: Perceived patient care outcome and recruitment - multivariate linear regression models

	Model A (Recruitment without deleted item)					Model B (Recruitment with one deleted item)				
	Standardized Coefficients Beta	t	*p*	95% Confidence Interval for B		Standardized Coefficients Beta	t	*p*	95% Confidence Interval for B	
				Lower Bound	Upper Bound				Lower Bound	Upper Bound
(Constant)		5.147	0.000	18.159	40.631		6.010	0.000	21.281	41.989
Profession (x)	0.181	2.638	0.009	0.864	5.939	0.198	3.035	0.003	1.287	6.027
Recruitment (y2)	-0.062	-0.766	0.444	-0.446	0.196	-0.027	-0.347	0.728	-0.378	0.264
x*y2	0.007	0.099	0.921	-0.366	0.405	0.017	0.251	0.802	-0.331	0.428
Sex	-0.146	-2.581	0.010	-6.041	-0.815	-0.148	-2.721	0.007	-5.815	-0.936
Age	0.075	1.332	0.184	-0.041	0.212	0.100	1.826	0.069	-0.009	0.230
Education	-0.002	-0.031	0.975	-1.594	1.545	-0.017	-0.275	0.783	-1.272	0.960
Outpatient unit	-0.024	-0.248	0.804	-4.561	3.541	-0.017	-0.183	0.855	-4.283	3.554
Emergency unit	0.063	0.666	0.506	-2.725	5.515	0.078	0.852	0.395	-2.246	5.677
Surgical unit	-0.088	-1.240	0.216	-8.003	1.816	-0.078	-1.148	0.252	-7.586	1.995
Paediatric unit	-0.065	-1.048	0.296	-8.641	2.636	-0.055	-0.924	0.356	-8.110	2.927
Logistic unit	-0.131	-1.290	0.198	-6.525	1.358	-0.112	-1.132	0.259	-5.995	1.616
Empowerment	0.243	3.985	0.000	0.158	0.467	0.228	3.816	0.000	0.139	0.436
Commitment	0.123	2.242	0.026	0.031	0.477	0.116	2.219	0.027	0.027	0.452
Trust	0.282	4.337	0.000	0.261	0.694	0.258	4.078	0.000	0.222	0.636

Information sharing (y5)

A2: Perceived patient care outcome and information sharing - multivariate linear regression models

	Model A (Information Sharing without deleted item)					Model B (Information Sharing with three deleted items)				
	Standardized Coefficients Beta	t	*p*	95% Confidence Interval for B		Standardized Coefficients Beta	t	*p*	95% Confidence Interval for B	
				Lower Bound	Upper Bound				Lower Bound	Upper Bound
(Constant)		6.329	0.000	21.462	40.827		6.542	0.000	20.926	38.921
Profession (x)	0.180	2.619	0.009	0.839	5.905	0.208	3.171	0.002	1.455	6.211
Information Sharing (y5)	0.226	3.149	0.002	0.211	0.914	-0.193	-2.780	0.006	-1.018	-0.174
x*y5	-0.166	-2.414	0.016	-1.082	-0.110	0.147	2.179	0.030	0.062	1.216
Sex	-0.128	-2.287	0.023	-5.640	-0.424	-0.150	-2.793	0.006	-5.841	-1.013
Age	0.056	1.003	0.317	-0.062	0.192	0.085	1.559	0.120	-0.025	0.212
Education	-0.010	-0.154	0.877	-1.686	1.440	-0.014	-0.233	0.816	-1.235	0.973
Outpatient unit	-0.008	-0.083	0.934	-4.103	3.772	0.006	0.065	0.948	-3.736	3.993
Emergency unit	0.036	0.389	0.698	-3.247	4.847	0.065	0.716	0.474	-2.478	5.318
Surgical unit	-0.085	-1.217	0.225	-7.801	1.839	-0.077	-1.147	0.252	-7.506	1.978
Paediatric unit	-0.076	-1.252	0.212	-9.058	2.015	-0.060	-1.023	0.307	-8.296	2.618
Logistic unit	-0.113	-1.152	0.250	-6.067	1.586	-0.092	-0.947	0.344	-5.533	1.936
Empowerment	0.213	3.644	0.000	0.126	0.423	0.224	3.926	0.000	0.141	0.425
Commitment	0.175	3.076	0.002	0.129	0.586	0.153	2.800	0.005	0.094	0.539
Trust	0.239	4.039	0.000	0.208	0.602	0.256	4.523	0.000	0.240	0.609

Job Quality measurement (y6)

A3: Perceived patient care outcome and job quality - multivariate linear regression models

	Model A (Job Quality without deleted item)					Model B (Job Quality with one deleted item)				
	Standardized Coefficients Beta	t	*p*	95% Confidence Interval for B		Standardized Coefficients Beta	t	*p*	95% Confidence Interval for B	
				Lower Bound	Upper Bound				Lower Bound	Upper Bound
(Constant)		6.887	0.000	25.055	45.098		6.902	0.000	24.601	44.220
Profession (x)	0.160	2.370	0.018	.509	5.485	0.140	2.082	0.038	0.143	5.086
Job Quality (y6)	0.204	2.790	0.006	.248	1.434	0.171	2.400	0.017	0.142	1.431
x*y6	-0.125	-1.782	0.076	-1.489	.074	-0.100	-1.521	0.129	-1.526	0.196
Sex	-0.143	-2.542	0.011	-5.936	-.757	-0.141	-2.501	0.013	-5.895	-0.703
Age	0.084	1.515	0.131	-.029	.221	0.080	1.432	0.153	-0.034	0.216
Education	-0.007	-0.114	0.909	-1.642	1.462	0.031	0.483	0.629	-0.902	1.489
Outpatient unit	-0.046	-0.495	0.621	-4.936	2.951	-0.057	-0.614	0.540	-5.202	2.728
Emergency unit	0.027	0.292	0.770	-3.459	4.666	0.020	0.208	0.835	-3.644	4.506
Surgical unit	-0.078	-1.112	0.267	-7.575	2.103	-0.096	-1.368	0.172	-8.183	1.472
Paediatric unit	-0.069	-1.125	0.262	-8.725	2.379	-0.073	-1.198	0.232	-8.943	2.176
Logistic unit	-0.157	-1.584	0.114	-6.933	.748	-0.167	-1.678	0.094	-7.142	0.566
Empowerment	0.211	3.542	0.000	.120	.421	0.207	3.470	0.001	0.115	0.417
Commitment	0.120	2.195	0.029	.025	.464	0.121	2.200	0.029	0.026	0.466
Trust	0.223	3.789	0.000	.182	.574	0.218	3.627	0.000	0.169	0.571

Appendix 2. Results of SEM-PLS modelling

We performed structural equation modelling with partial least squares (SEM-PLS) using SmartPLS 3, testing the pathway models between the high performance work system (HPWS) and perceived patient care outcomes (Figure 1). The path coefficients were estimated using consistent PLS algorithm (connecting all LVs for initial calculation).

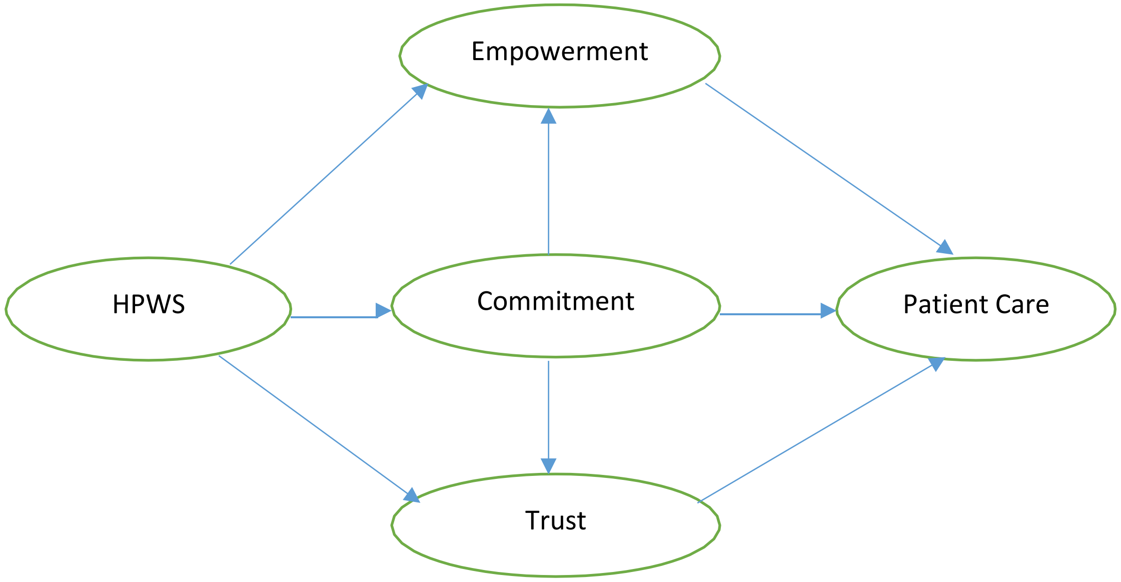

Figure 1. Pathway model between HPWS and perceived patient care outcomes

We examined the differences between doctors and nurses through two approaches: (1) model differences between doctors and nurses; (2) moderation effects of occupation (doctors vs nurses) on the effects of the three mediating factors: empowerment, commitment and trust on perceived patient care outcomes.

The group comparisons revealed that differences existed between doctors and nurses in the tested pathways between HPWS and perceived patient care outcomes, especially over the path “HWPS -> Commitment -> Quality of Care” and the path “Trust -> Quality of Care” (Table 1). “Commitment” appeared to have a greater effect on doctors; whereas “Trust” had a greater effect on nurses. Statistical significance appeared in the differences in several pathway coefficients of the “Teams” models: doctors had a greater coefficient in “Commitment -> Quality of Care” and “Teams -> Trust”; whereas nurses had a greater coefficient in “Commitment -> Trust”.

Table 1. Pathway model comparisons between doctors and nurses

	Job Security			Recruitment			Training			Transformational Leadership			Information Sharing			Job Quality			Teams		
Path Coefficient	Doctor	Nurse	p value of Difference	Doctor	Nurse	p value of difference	Doctor	Nurse	p value of difference	Doctor	Nurse	p value of difference	Doctor	Nurse	p value of difference	Doctor	Nurse	p value of difference	Doctor	Nurse	p value of difference
Commitment -> Empowerment	**0.529**	**0.526**	0.974	**0.493**	**0.444**	0.619	**0.585**	**0.534**	0.59	**0.543**	**0.503**	0.664	**0.610**	**0.534**	0.406	**0.550**	**0.525**	0.792	**0.517**	**0.574**	0.538
Commitment -> Quality of Care	0.146	-0.124	**0.04**	0.152	-0.119	**0.038**	0.162	-0.126	**0.024**	0.154	-0.131	**0.03**	0.123	-0.131	0.057	0.152	-0.128	**0.032**	**0.165**	-0.126	**0.028**
Commitment -> Trust	**0.353**	**0.469**	0.285	**0.306**	**0.277**	0.78	**0.394**	**0.365**	0.766	**0.322**	**0.384**	0.501	**0.377**	**0.471**	0.352	**0.412**	**0.480**	0.49	**0.329**	**0.539**	**0.037**
Empowerment -> Quality of Care	**0.383**	**0.293**	0.403	**0.379**	**0.287**	0.41	**0.375**	**0.306**	0.526	**0.379**	**0.311**	0.527	**0.398**	**0.305**	0.368	**0.377**	**0.308**	0.534	**0.372**	**0.297**	0.495
HPWS -> Commitment	**0.502**	**0.400**	0.272	**0.473**	**0.543**	0.399	**0.282**	**0.475**	0.065	**0.354**	**0.366**	0.901	**0.425**	0.552	0.752	**0.358**	**0.335**	0.821	**0.473**	**0.357**	0.238
HPWS -> Empowerment	0.138	**0.131**	0.941	**0.223**	**0.242**	0.854	0.058	0.084	0.807	**0.156**	**0.186**	0.77	-0.054	0.046	0.402	**0.140**	**0.150**	0.92	**0.178**	0.015	0.136
HPWS -> Trust	**0.300**	**0.239**	0.645	**0.415**	**0.536**	0.21	**0.360**	**0.439**	0.438	**0.479**	**0.516**	0.681	**0.300**	0.193	0.488	**0.242**	**0.284**	0.68	**0.346**	0.089	**0.039**
Trust -> Quality of Care	0.124	**0.368**	0.083	0.120	**0.366**	0.076	0.118	**0.362**	0.082	0.119	**0.364**	0.075	0.127	**0.369**	0.096	0.123	**0.363**	0.084	0.119	**0.369**	0.085

Note: **Bold** indicates statistical significance ($p<0.05$)

Occupation had a significant moderation effect on the link between “Trust” and perceived “Quality of Care” (Figure 2, 3, 4, 5, 6, 7, 8). No significant moderation effects were detected between “Empowerment” and perceived “Quality of Care”, or between “Commitment” and perceived “Quality of Care”.

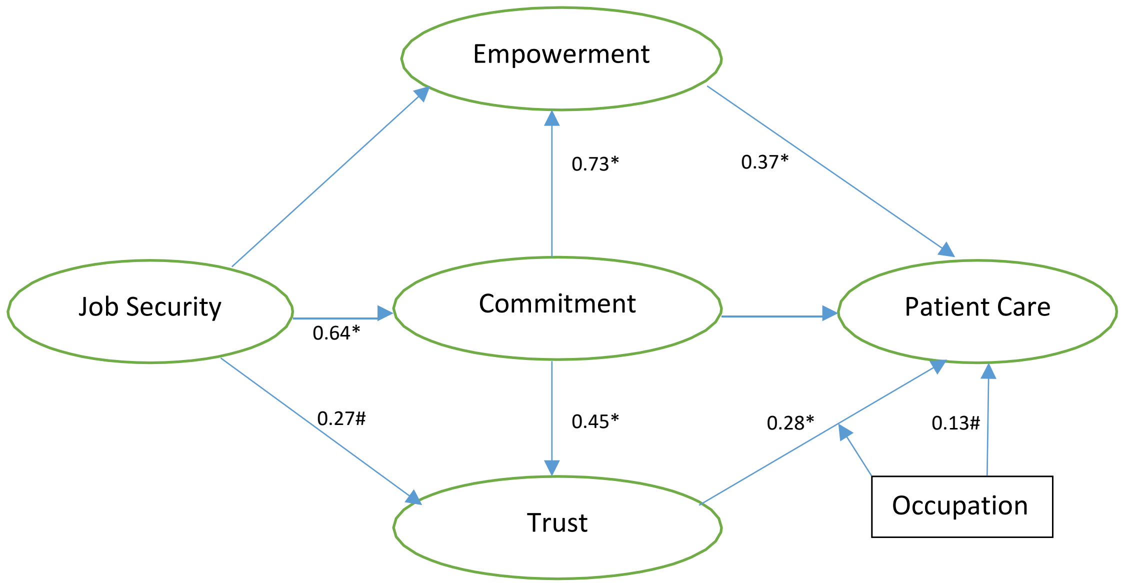

Figure 2. Pathway between job security and patient care outcomes (# p<0.05; *p<0.01)

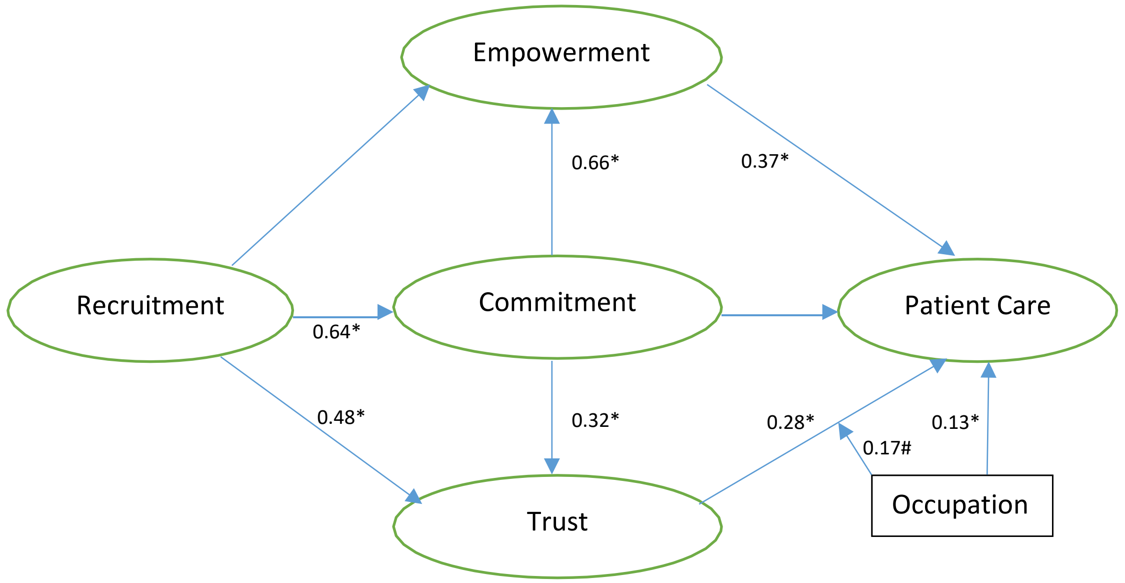

Figure 3. Pathway between recruitment and patient care outcomes (# p<0.05; *p<0.01)

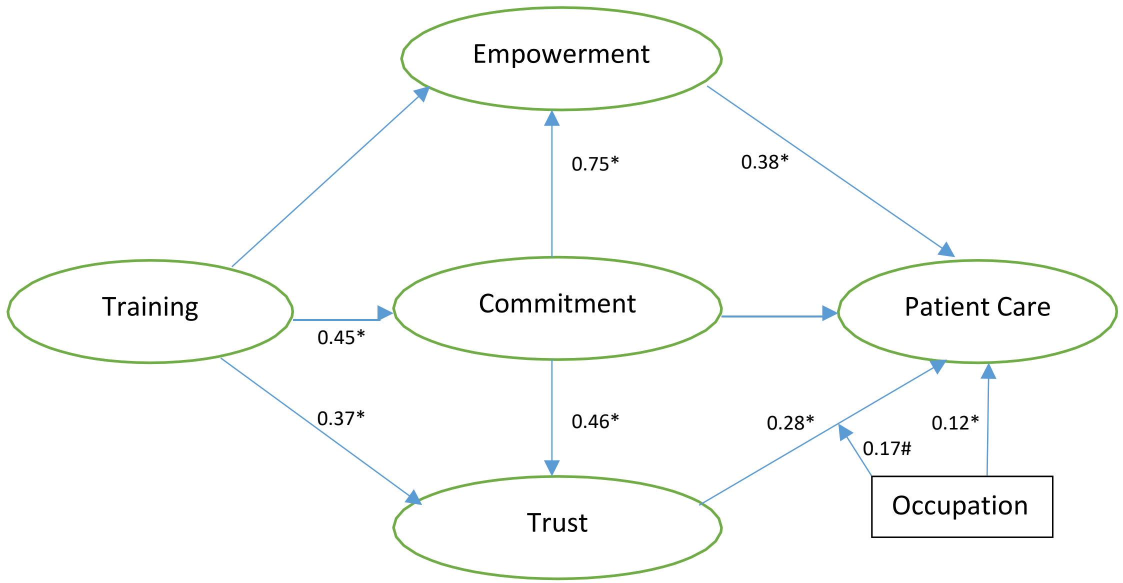

Figure 4. Pathway between training and patient care outcomes (# p<0.05; *p<0.01)

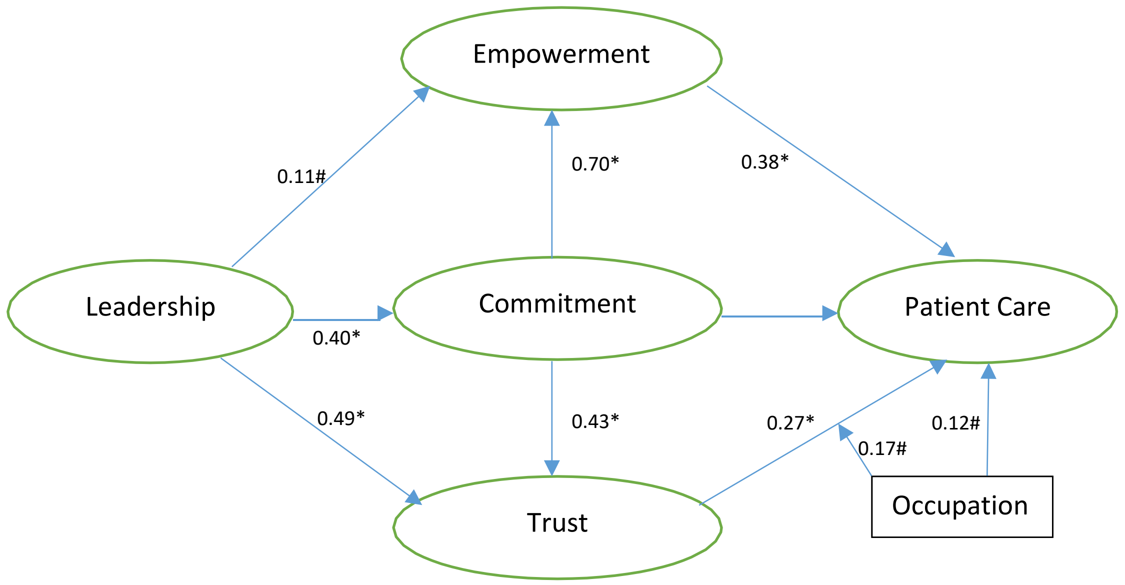

Figure 5. Pathway between transformational leadership and patient care outcomes (# p<0.05; *p<0.01)

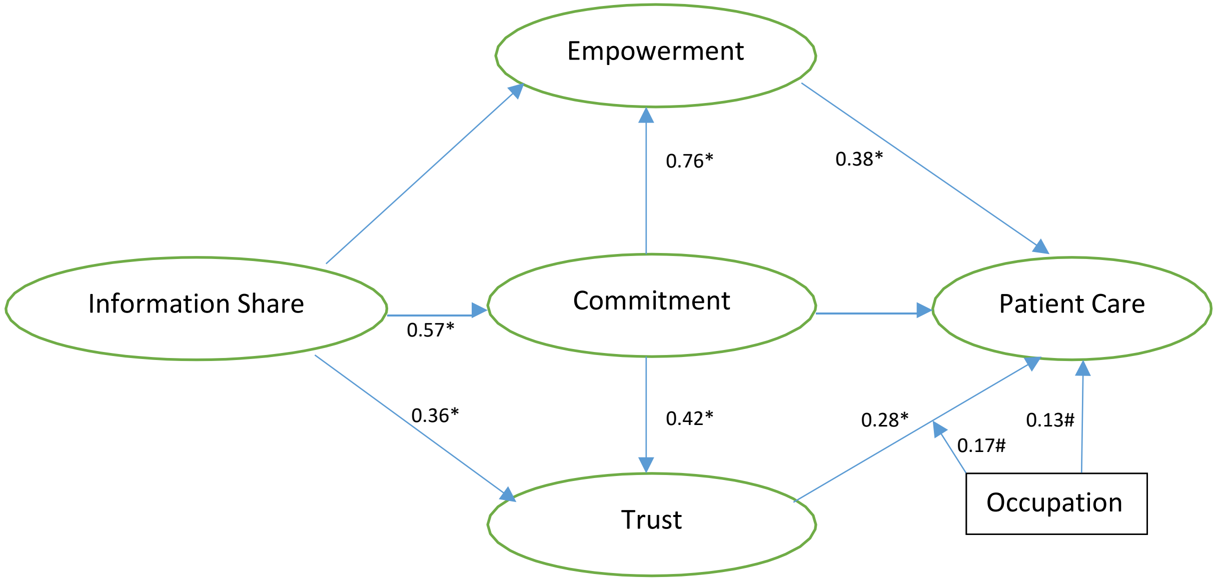

Figure 6. Pathway between information sharing and patient care outcomes (# p<0.05; *p<0.01)

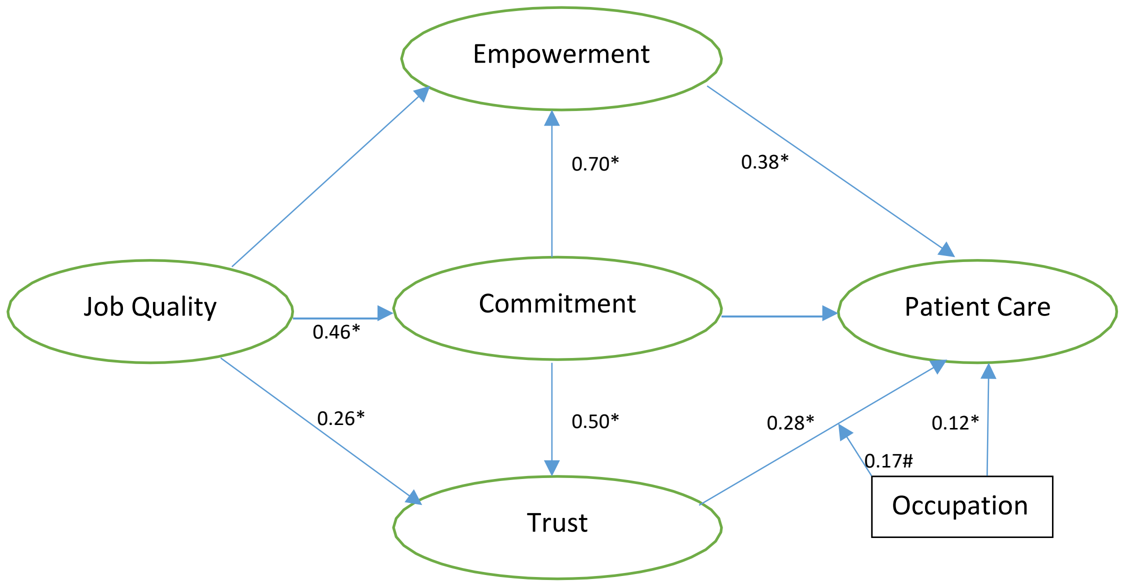

Figure 7. Pathway between job quality and patient care outcomes (# p<0.05; *p<0.01)

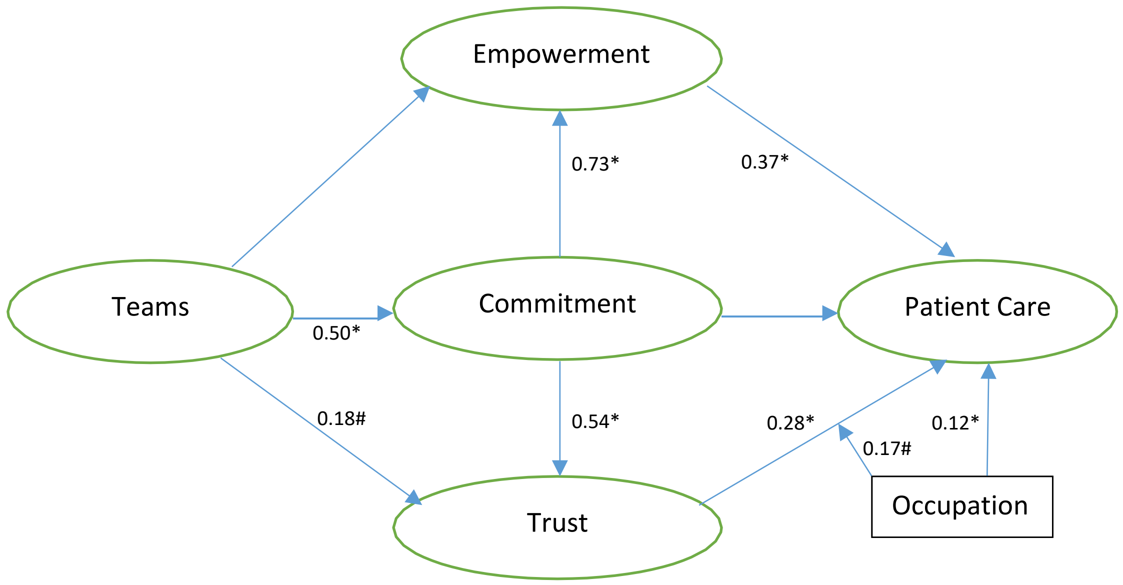

Figure 8. Pathway between teams and patient care outcomes (# p<0.05; *p<0.01)
